# Strategies in supporting inclusive education for autistic students—A systematic review of qualitative research results

**DOI:** 10.1177/23969415221123429

**Published:** 2022-09-21

**Authors:** Linda Petersson-Bloom, Mona Holmqvist

**Affiliations:** 225280 Faculty of Learning and Society, Malmö University, Malmö, Sweden; The National Agency for Special Needs Education and School (SPSM), Sweden; 225280 Faculty of Learning and Society, Malmö University, Malmö, Sweden

**Keywords:** Autism, inclusive education, strategies in the learning environment, qualitative research

## Abstract

**Background and Aim:**

Strategies to modify and adjust the educational setting in mainstream education for autistic students are under-researched. Hence, this review aims to identify qualitative research results of adaptation and modification strategies to support inclusive education for autistic students at school and classroom levels.

**Method:**

In this systematic review, four databases were searched. Following the preferred PRISMA approach, 108 studies met the inclusion criteria, and study characteristics were reported. Synthesis of key findings from included studies was conducted to provide a more comprehensive and holistic understanding.

**Main Contribution:**

This article provides insights into a complex area via aggregating findings from qualitative research a comprehensive understanding of the phenomena is presented. The results of the qualitative analysis indicate a focus on teachers' attitudes and students' social skills in research. Only 16 studies were at the classroom level, 89 were at the school level, and three studies were not categorized at either classroom or school level. A research gap was identified regarding studies focusing on the perspectives of autistic students, environmental adaptations to meet the students' sensitivity difficulties, and how to enhance the students' inclusion regarding content taught and knowledge development from a didactic perspective.

**Conclusions and Implications:**

Professional development that includes autism-specific understanding and strategies for adjusting and modifying to accommodate autistic students is essential. This conclusion may direct school leaders when implementing professional development programs. A special didactical perspective is needed to support teachers' understanding of challenges in instruction that autistic students may encounter.

## Introduction

Based on the Salamanca Statement (1994), children with special educational needs and disabilities (SEND) should have access to inclusive education in general schools that are adapted to meet a diverse range of educational needs. Furthermore, The United Nations (UN) Convention on the Rights of Persons with Disabilities, Article 24 (Convention on the Rights of Persons with Disabilities, 2008), states that people with disabilities should receive the support they need to achieve an effective education and that effective individual support should be provided to maximize academic and social development, as well as United Nation's agenda 2030 Goal 4.5 which demands “equal access to all levels of education and vocational training for the vulnerable, including persons with disabilities, indigenous peoples and children in vulnerable situations” (UN General Assembly, 2015). This has resulted in an increased prevalence of autistic students in ordinary classroom education during the last decade (Maenner et al., 2020). The educational sector has to be prepared to educate autistic students in general school settings to a higher degree than before (Parsons et al., 2011). Unfortunately, research has had a strong focus on research *about* inclusion rather than research *in* inclusive education (Susinos & Parrilla, 2013). Even if both aspects are important, characteristics of qualitative studies in inclusive settings unveil the voices of students and parents and support ecological validity (Ledford et al., 2016) when capturing aspects of importance of what needs autistic students have in the school-context. Combining and making sense of data from several smaller qualitative studies goes beyond the understanding of each single study, and results from several different studies conducted in various context can strengthen each study's findings (Soilemezi & Linceviciute, 2018). Echeita et al. (2021) claim that qualitative research has the strength to give the students, and their families, respect to their voices by listening to their view of how inclusive education is experienced from their viewpoint and in what way they feel included in the school context. Even if almost 30 years have passed since the Salamanca Statement, for some reason it has been difficult to develop inclusive educational settings meeting the needs of autistic students. Goodall (2019) presents that in Ireland, it is stated that mainstream education is the best placement for autistic students. However, capturing the view of autistic teenage boys it is suggested that they perceive themselves as better supported in alternative education provision, which is identified in the students’ evaluation and their knowledge development (Goodall, 2019). Furthermore, Merry (2020), as well as Waddington and Reed (2017) support this finding and argue that students in regular schools do not automatically show greater academic development compared to autistic students in specialist placements. To provide society with research results to enhance the quality of inclusive education for autistic students, we suggest focusing on qualitative research to deepen and personalize the understanding of how inclusive education is experienced by the students. Research has tended to focus more on how autistic students can develop and change to enhance their possibilities to be included in a regular school environment, as opposed to examining what can be developed and changed in the learning environment (Crosland & Dunlap, 2012; Osborne & Reed, 2011). As autism is a life-long condition, the strategies to adjust the educational setting to support it in a better way are a key aspect for creating inclusive education. But how and what is still not answered, which this systematic review addresses.

### Present study

Several previous systematic reviews, mainly synthesizing quantitative research results, have focused on different types of interventions intended to develop skills in autistic individuals, enhancing their capability to handle mainstream education (Bond et al., 2016; Watkins et al., 2017). The present systematic review instead focuses on environmental strategies (modifications and adaptations) at the school and classroom level to support the inclusive education of autistic students. There are two systematic reviews, which have focused on the physical environment, how to design a classroom to support autistic students (Martin, 2016), and if modifications improve the physical environment (Dargue et al., 2021). Furthermore, two recently published systematic reviews targeted interventions for peers of autistic students, aiming to change peers’ attitudes (Cremin et al., 2020; Morris et al., 2021). A recently published systematic review investigated the effectiveness of school-related outcomes of interventions for autistic students (Macmillan et al., 2021). In Leifler et al. (2020), which included quantitative studies, accommodations to the learning environment were at focus, concluding that it is a promising area, but one that needs to be studied further.

This review differs from the previous by its focus on research studies using qualitative methods, capturing the respondents’ views and situations at a detailed level based on their experiences.

The rationale for this is based on the argument that qualitative studies in a review add to the comprehensive understanding of the phenomena (Tong et al., 2016) to generate a broader insight regarding the perspective of the participants (Chong et al., 2018). In related research fields, it has been claimed that qualitative studies “have been especially useful in improving understanding of patient experiences and perspectives” (Cohen & Gooberman-Hill, 2019, p. 2). Furthermore, synthesizing qualitative research makes results more substantial, as they include a greater variety of both participants and descriptions (Sherwood, 1999). As such, this study focuses on students, teachers, and parents to better understand their experiences. Supporting the decision to synthesize qualitative research findings, Thomas and Harden (2008) underline the value of examining a more complex view: “The act of seeking to synthesize qualitative research means stepping into more complex and contested territory than is the case when only RCTs are included in a review” (p. 2).

### Aim and research questions

This systematic review aimed to summarize and synthesize key findings of research results from qualitative analyses, focusing on results of modifications and adaptations at school and classroom levels for autistic students in general educational settings, to support inclusive education. The following research questions were asked:

RQ 1: What results can be found regarding modifications and adaptations at school and classroom levels for autistic students?
RQ 2: In what way do the studies consider the students’, families’, and teachers’ viewpoints?
RQ3: Which gaps in can be found and addressed in future research?

## Method

### Background of the study

The search is based on a project, aiming to capture both quantitative and qualitative studies. Details about the project can be found in PROSPERO (number: 2019 CRD42019124496). The review conducted by Leifler et al. (2020) reports the studies with a quantitative approach. The review methodology in the joint project followed the steps in the Preferred Reporting Items for Systematic reviews and Meta-Analyses (PRISMA) statement (Moher et al., 2009). The search was conducted in collaboration with a group of seven researchers from three universities who collaborated in the process of defining the search strategy and, to some extent, the initial screening. The studies were divided into two research groups based on methodology, that is, quantitative or qualitative methods, and this article reports the results of the qualitative analysis. However, during the search the team worked jointly in the first screening stages.

### Search strategy

The search process started in September 2018 with the identification of possible search vocabularies, with test searches carried out in October 2018. The final searches were performed by two librarians, at the Karolinska Institute University Library in November-December 2018 (the full search strategy is provided as Supplementary Material search strategy). The literature search was performed using the following databases: ERIC (ProQuest), Medline (Ovid), PsycINFO (Ovid), and Web of Science. Journal articles published in English between 1990 and 2018 were included.

### Screening

**Figure 1.**
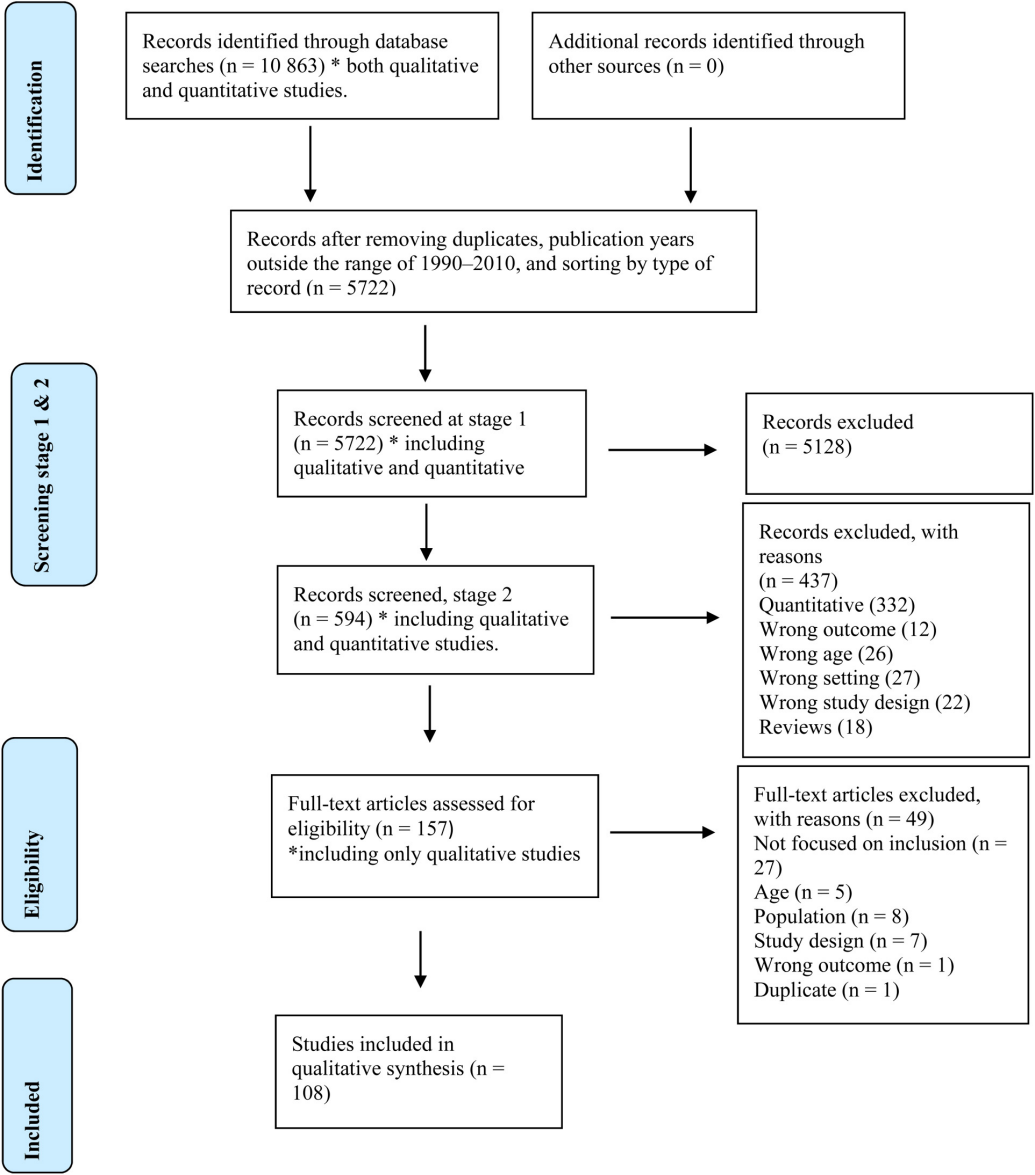
PRISMA flow chart.

The database searches retrieved 10,863 records, as shown in the PRISMA flow chart (Moher et al., 2009) in Figure 1.

Titles and abstracts from 5,722 records were assessed according to the eligibility criteria by two independent reviewers. The records were screened according to the inclusion and exclusion criteria (Table 1).

**Table 1.** Inclusion and exclusion criteria.

Inclusion criteria	Exclusion criteria
Students with ASD aged 5 to 19 years attending mainstream schools	Non-mainstream school settings (e.g., segregated provision or clinical setting) with participants below 5 years and above 19 years of age
Interventions in the learning context (social, pedagogical, and physical) including: organization, collaboration, teacher competence, peer involvement, and interventions specifically focusing on school subjects or other activities in school.	Strategies and interventions that target solely personal changes in a child (e.g., skill training)
	Reviews, theses, protocols, book reviews

#### Pre-screening for identification of approaches

After the first screening stage (Figure 1), 594 articles remained. A second screening process was conducted (stage 2) to divide the articles into groups according to whether they had quantitative or qualitative methodological approaches. At this stage, some articles were found to not meet the requirements of the inclusion criteria, thus resulting in the exclusion of 332 articles with quantitative methodologies and 105 articles for different reasons, as reported in Figure 1. The full texts of the articles with qualitative methodologies that remained after abstract screening were then acquired.

### Full-text review

As mentioned in PROSPERO, the authors of this article were responsible for analyzing the articles with a qualitative research approach. We independently screened the full texts of the articles using the same inclusion criteria that were used in the abstract screening process.

This screening led to a consensus regarding 114 articles to be included, 21 to be excluded, and 22 articles on which the reviewers disagreed regarding inclusion. Cohen's kappa was calculated, and the results showed *moderate agreement* (0.57). The articles which were disagreed upon were discussed until a resolution was achieved, which resulted in 5 more articles being added, to bring the total to 119 articles. Finally, after carefully reading the full text of each article, 11 more articles were excluded because they either did not meet the inclusion criteria or did not fulfill the qualitative checklist of the Critical Appraisal Skills Program (2018) (Nadelson & Nadelson, 2014). All the excluded articles are reported in Figure 1 (eligibility). Thus, 108 articles were included for data extraction and synthesis.

### Data extraction and data synthesis

Data were extracted using a standardized form designed by the authors in collaboration and included the following information: (a) author and publication year; (b) country in which the research was conducted; (c) diagnosis; (d) special educational needs and disabilities (SEND); (e) grades; (f) participants (students with ASD, peers, parents, teachers, paraprofessionals, and other school professionals); (g) method; (h) research focus; and (i) result/outcome. A template was designed, to increase the inter-rater reliability of coded data extraction. Each aspect above (a to i) had its own column, where information was inserted to give a sound overview of the included articles’ content. The descriptive coding (Saldaña, 2014) of items (a) to (i) was carried out by the first author and checked for reliability by the second author. In qualitative systematic reviews, a variety of different methods is often used in conducting the synthesis (Barnett-Page & Thomas, 2009). We integrated the results from each study via a qualitative text analysis of the results of the included articles. In Barnett-Page and Thomas (2009), textural narrative synthesis includes study characteristics, context, and results being compared across studies. Further, as posited in Harden et al. (2004) structured summarizations can be developed and put into a wider context. For this, a thematic synthesis (Thomas & Harden, 2008) of the studies’ results (i) was made by the second author, and checked for reliability by the first author. The thematic syntehsis aimed to capture the areas if interest in each study, and coding studies with the same focus into categories where the studies’ results where synthesized. In total, four categories of research focus were identified; Didactical perspective, Attitudes and views, Environmental sensitivity, and Social skills focus. Furthermore, in each category, the findings were divided into two levels; school and classroom.

## Results

The results are presented in two sections. In the first section the characteristics of the included studies are elaborated upon and in the second section key findings from the synthesis are presented. The references used in the synthesis are visualized within brackets as numbers and in a table illustrating authors and publication years (Table 2). The descriptive analysis is fully reported in Supplementary Material (Descriptive Characteristics of Articles Synthesized).

**Table 2.** References used visualized in the brackets with numbers.

Nr	Author/year
1	Able et al. (2015)
2	Anglim et al. (2018)
3	Asaro and Saddler (2009)
4	Ashbee and Guldberg (2018)
5	Ashby (2010)
6	Barned et al. (2011)
7	Beecher and Darragh (2011)
9	Bond and Hebron (2016)
10	Bond et al. (2017)
11	Bottema-Beutel et al. (2016)
13	Bradley (2016)
14	Breivik and Hemmingsson (2013)
16	Carter et al. (2014)
17	Cook et al. (2018)
18	Corkum et al. (2014)
19	Daniel and Billingsley (2010)
20	Dann (2011)
21	Dean et al. (2013)
22	Dillon et al. (2016)
24	Eldar et al. (2010)
25	Emam and Farrell (2009)
27	Ezzamel and Bond (2017)
28	Finch et al. (2013)
30	Frederickson et al. (2010)
31	Gentry and Lindsey, (2012)
32	Glashan et al. (2004)
33	Grenier and Yeaton (2011)
34	Guldberg et al. (2017)
37	Hedges et al. (2014)
38	Higginson & Chatfield, (2012)
39	Howe and Stagg (2016)
40	Humphrey and Lewis (2008b)
41	Humphrey and Symes (2010)
42	Jindal- Snape et al. (2005)
43	Jones and Block (2006)
47	Keane et al. (2012)
48	Kucharczyk et al. (2015)
49	Lamb et al. (2016)
52	Lewis et al. (2005)
53	Lindsay et al. (2013)
54	Lindsay et al. (2014)
57	McCollow et al. (2013)
58	Macdonald et al. (2017)
59	Maher (2017)
61	Marks et al. (2000)
63	McAllister and Sloan (2016)
64	McAllister and Maguire (2012a)
65	McAllister and Maguire (2012b)
67	Moyse and Porter (2015)
70	Oakley et al. (2013)
71	Ochs et al. (2001)
73	Pfeiffer et al. (2019)
74	Poon et al. (2014)
75	Poonam (2014)
77	Ravet (2018)
79	Rosetti and Goessling (2010)
80	Saggers et al. (2011)
81	Saggers (2015)
83	Sansosti and Sansosti (2012)
84	Santarosa and Conforto (2016)
85	Sciutto et al. (2012)
90	Soto-Chodiman et al. (2012)
91	Starr and Foy (2010)
92	Starr et al. (2016)
93	Stokes et al. (2017)
94	Symes and Humphrey (2011a)
95	Symes and Humphrey (2011b)
96	Symes and Humphrey (2012)
97	Taneja Johansson (2014)
99	Tissot (2011)
100	Tobias (2009)
101	Tso and Strnadová (2017)
104	Whalon and Hart (2011)
105	Wong (2017)
106	Woodfield and Ashby (2016)
107	Young et al. (2017)

### Characteristics of included studies

In terms of provenance, most of the 108 articles were from English-speaking countries. This mirrors the databases used (English was the main language). In total, articles from 17 different countries were captured during the search (Table 3).

**Table 3.** Origin of research based on country.

Country	N
USA	37
UK	34
Australia	12
Canada	5
Ireland	3
New Zealand	3
Hong Kong	2
Northern Ireland	2
Spain	2
Brazil	1
India	1
Israel	1
Palestine	1
Portugal	1
Singapore	1
Sweden	1
Zimbabwe	1

#### Autistic students’ characteristics

The majority of the articles focused on students with typical learning development, excluding students with learning difficulties or intellectual disabilities (ID) in combination with autism. In total 39 of the studies included students from school grades in primary school. Secondary school is represented in 34 of the included studies. In 20 of the studies all school grade levels are included, indicating a more general and school-level research focus. In total, 70 articles included students from more than one grade level, while 34 studies examined only one explicit grade level.

#### Participants

In total, 61 articles included multiple participants’ perspectives on education outcomes. Teachers and other school staff (e.g., para-professionals) were represented in 80 of the included articles. In 22 studies, teachers’ perspectives were in focus, and in five, the perspective was that of school officials (paraprofessionals, principals, and other school staff). In total, 12 of the articles included a mix of staff members (e.g., teachers and other school staff).

A smaller number, 12 of the articles, focused only on the perspectives of autistic students [3, 11, 39, 40, 41, 49, 63, 74, 80, 81, 96, 105]. Data collected from autistic participants in combination with other participants’ perspectives were included in 46 studies. In total 33 of the articles included parents in different combinations. Five of these centered solely on parents. Peers were at focus in four of the articles, and with a mix of autistic students in three of the included articles. The school grade level of included peers ranges from primary to secondary school.

#### Research method

The methods used were mainly interviews; 66 articles used interviews to collect data. Case studies were implemented in 26 articles, questionnaires were qualitatively analyzed (mainly regarding attitudes) in 19 articles, observations in 12 studies, and a mixed-methods approach in 9 articles. In some articles, more than one method was used, but not as part of a mixed-methodology approach.

#### Research focus

Four categories of research focus were identified; Attitudes and views, Didactical perspective, Environmental sensitivity, and Social skills focus. The initial analysis indicated a majority of studies focusing on attitudes (e.g., experiences and views regarding inclusive teaching for autistic students). The outcomes in such studies are often teachers’ or other stakeholders increased attitudes towards inclusive education. The majority of the studies (80) included the views or attitudes of teachers or paraprofessionals. Teachers’ attitudes and views regarding inclusive education were found to be crucial for the success of inclusive strategies. In some studies, these were identified as one of the most important factors affecting whether or not autistic students reported positive experiences regarding inclusive education (e.g., Higginson & Chatfield, 2012), and whether the teachers accepted the autistic students (Reupert et al., 2015).

Regarding communication and social relationships, studies have reported how acceptance can be strengthened. In 36 articles, some aspects of social or communicative training or attitudes were examined by studying the social situation of autistic students (Hay & Winn, 2005; Humphrey & Lewis, 2008b) or social relationships and friendships (e.g., Calder et al., 2013; Cook et al., 2018; Daniel & Billingsley, 2010; Jones & Howley, 2010). As they often have altered social and communicative abilities, this focus might contradict the attitude of accepting students’ difficulties as a result of the education situation.

### Key findings synthesized: Strategies for inclusive education

The results of the reviewed studies are in total summarized in Supplementary Material (Categories and Research Results). Articles not referred to directly in the text are presented as references in the Supplementary Material (References, not referred to in the text). The key findings will be presented at the school and classroom levels.

#### School level

In total, 89 studies had a school-level perspective of inclusive education, and all of them focused on students’ social skills (13) or attitudes and views (52). In 24 articles, both perspectives were included.

A major result was limited professional development and knowledge about autism as a challenge and limitation for inclusive education, which was found in 31 studies [2, 4, 5, 6, 16, 18, 22, 24, 28, 30, 32, 34, 40, 42, 53, 54, 57, 59, 67, 74, 77, 83, 85, 91, 92, 94, 97, 99, 100, 101, 107]. Furthermore, the challenges with autistic symptoms and meeting the needs of the students were found to be difficult for teachers to handle in a traditional mainstream class, depending on the severity of autism, which was reported in eight studies [16, 18, 24, 25, 40, 48, 53, 90]. In the studies by Humphrey and Lewis (2008a, 2008b) and Kucharczyk et al. (2015), this was also supported by data from students. Moreover, Ashby (2010) found that pupils with disabilities felt alienated in the classroom and that the teachers downplayed their differences.

After participating in professional development on autism, teachers were reported to show more positive attitudes regarding inclusive education [7, 9, 10, 38, 75]. Barned et al. (2011) identified that attitudes were related to the severity of the challenges faced by the children; for example, for children with more severe disabilities, teachers had more negative attitudes regarding inclusive schooling. A similar result was also reported by Carter et al. (2014), where students’ characteristics were identified as barriers to inclusive education. Furthermore, Barned et al. (2011) determined that even if the teachers had positive attitudes, they sometimes misunderstood what autism constitutes. Another study (Bond et al., 2017) found increased self-efficacy in teachers after participating in a professional development program focusing on autism. Various studies have indicated the need for professional development regarding inclusive education to help teachers cope with the challenges associated with teaching autistic students (e.g., Corkum et al., 2014; Young et al., 2017). Having investigated teaching assistants, Emam and Farrell (2009) established that they play an important role for both teachers and autistic students, as the former often rely on them to make the classroom situation work.

The results show that although autistic students wish to have friends, they have difficulties maintaining friendships and understanding the conditions for friendship (e.g., Daniel & Billingsley, 2010; O’Hagan & Hebron, 2017). The results highlight the role peers play in inclusive education for autistic students and accepting them [1, 11, 27, 41, 61, 71, 79, 81]. Furthermore, the relationship between parents and teachers is revealed as crucial for positive inclusive education (e.g., Bottema-Beutel et al., 2016; Lindsay et al., 2014).

### Classroom level

Although the studies at the school level have implications for the classroom, only a small number of the articles (16 of the 108) were based on data collected at the classroom level. Six of the studies investigated environmental sensitivity to better understand the difficulties the students faced regarding participation in classroom activities [39, 63, 64, 65, 73, 84]. As a prominent aspect of autism is difficulties in perception and sensory processing, only six studies are insufficient to contribute adequate knowledge to address this challenge. The research results identify that the environment becomes an issue for the students learning. There is a need for adjusting the school environment—for example, sound and light aspects (e.g., McAllister & Sloan, 2016; Pfeiffer et al., 2019), which is important to enhance the students’ well-being in the school environment. Still, not enough knowledge is provided regarding a well-founded basis for decisions on how to design learning environments. Howe and Stagg (2016) conducted a study on the sensory experiences of autistic students to enable changes in the school environment that could decrease the negative impact on learning. They found that autistic students experienced various sensory challenges that affected their ability to learn. Pfeiffer et al. (2019) investigated the use of noise-attenuating headphones by autistic students and determined that their participation increased in various contexts, such as at home, at school, and in the community. To examine the potential factors that are important in an autism-friendly classroom, McAllister and Maguire (2012a, 2012b) examined the use of a Classroom Design Kit to make it easier for autistic students to identify what activities should be done in what locations, and to help them organize their schoolwork based on this knowledge. Exploring the use of laptops and tablets by autistic students for schoolwork, Santarosa and Conforto (2016) found that tablets are especially user-friendly tools.

Ten articles addressed content for knowledge development assessable for autistic students in inclusive education [3, 14, 31, 33, 43, 58, 70, 93, 96, 104].

Macdonald et al. (2017) claim that there is an urgent need for interventions to develop results that support autistic students in general education. They studied the implementation of an intervention model and conducted collaborative research (2017). Stokes et al. (2017) also focused on didactic strategies in the classroom. Their results showed how visual support, structure, concrete instruction, and timetables helped improve learning outcomes. In terms of strategies for physical education (PE), Grenier and Yeaton (2011) revealed that previewing the content of PE lessons with autistic students helped them prepare for participation during classes and that the students developed increased trust in the teacher as a result of this process. Researching the same area, Jones and Block (2006) found that visual support, as well as minimizing extra stimuli, and teacher collaboration improved the experience of autistic students. In terms of reading and writing, Asaro and Saddler (2009) established that self-regulated strategy development increased writing skills by enhancing students’ ability to plan and execute the writing process. Breivik and Hemmingsson (2013) also studied writing in adolescents with Asperger syndrome and found that using a computerized assistive technology device improved writing skills. This is in line with a study by Gentry and Lindsey Lindsey (2012), whose findings indicated that reading instructions could be adapted to the needs of autistic students via the use of assistive technologies. Multimodal reading (Oakley et al., 2013) was found to be supported by the use of information and communication technologies (ICT) and led to more positive attitudes towards reading in autistic students. Investigating how students experienced reading instructions, Whalon and Hart (2011) found that reading and language arts instruction did not always reflect or meet the individual needs of autistic learners. Symes and Humphrey (2012) compared student inclusion in different lesson groups and determined that autistic students were less frequently included in the lessons than students with dyslexia or those with no special educational needs. A positive learning and social inclusion were also more likely if the other students in the class had received an explanation and understood the diagnosis of the autistic student (Wastney et al., 2007).

### Research gaps and need for future research

Most studies have focused on teachers’ *attitudes* regarding inclusive education and understanding or developing social skills in autistic students. Although this is a very important aspect for powerful inclusive education, results of strategies to teach students with autism in general classrooms are rare. Ten studies were found with a didactical perspective, and by that, we identified a lack of studies that focuses on the didactical aspects of inclusive education. However, Macdonald et al. (2017) is an exception; their study of how to enhance the degree of knowledge development in students is important for raising the future knowledge development for autistic students in general classrooms. And as mentioned before, there is also a limited amount of studies (6) that address the role of sensory challenges in creating inclusive learning environments for autistic students.

Only one study included students with LDs, which reflects the view and limitations of what students we focus on in inclusive educational settings. In this field, students with a combination of autism and ID are not noticed by the found studies. The article which included an autistic student and LD (Jones & Block, 2006) was a study on the inclusivity of PE for an autistic girl.

Teacher assistants play an important role at the classroom level, and as there were only four [25, 83, 95, 97] studies that addressed their situation in some way, there is also a research gap in this area.

## Discussion

The objective of this systematic review was to summarize and synthesize key findings of research results from qualitative analyses, focusing on results of modifications and adaptations at school and classroom levels for autistic students in general educational settings, to support inclusive education. Here, both barriers and facilitators to develop inclusive education were detected. The results point towards a strong focus on strategies at the school level and foremost on implementing positive attitudes in teachers as a strategy to develop inclusive education for autistic students. Developing positive attitudes towards inclusive education is important, and there is a consensus that teacher attitudes are congruent with the effectiveness of inclusive education (Bolourian et al., 2021; Segall & Campbell, 2012) which we also identified. However, beyond positive attitudes, there is a need for finding strategies for the implementation of inclusive education. In agreement with previous research (e.g., Alexander et al., 2015; Bölte et al., 2021) this synthesis recognizes that not only positive attitudes are needed. A lack of professional development on autism understanding is identified as a barrier for inclusive education and is important for developing strategies to modify and adapt to the learning environment. The findings also support and justify previous systematic reviews focusing on the effect of peers on autistic students (Cremin et al., 2020; Morris et al., 2021).

It is important to mention that some studies found that teachers experienced autism traits and severity challenging to educate autistic students in a mainstream classroom (e.g., Humphrey & Lewis, 2008a, 2008b; Kucharczyk et al., 2015) which is a concern and could be a barrier to inclusive education.

The search in this systematic review only captured a few studies at the classroom level as the main source for data collection. As such, there is limited knowledge about how to change and adapt classroom instruction to meet the needs of autistic students. Furthermore, the studies did not focus on the students’ learning development from a didactic perspective. As schools in different parts of our world claim to support should both academic and social development, this is a serious lack of research focus (e.g., the Convention on the Rights of Persons with Disabilities).

Furthermore, the systematic review also aimed to investigate to what extent studies consider the students’, families’, and teachers’ viewpoints. The result indicates that the majority (80) of the included articles included the views of staff members, such as teachers and paraprofessionals. Far fewer studies (33) included the views of parents of autistic students. The view of autistic students is represented in combination with other participants’ views in a total of 46 studies. However, fewer (12) studies centered solely on the views of the autistic students themselves. The research gap of how autistic students perceive their school situation can be problematized with the perspective of the United Nations Convention on the Rights of the Child (1989). This is particularly notable in Article 12, which states that children have the right to be included in decisions regarding their lives. The lack of research including autistic students’ views on inclusion is also found in previous research (Fayette & Bond, 2018).

### Limitations

Several limitations can be acknowledged in this review. First, relevant studies may have been excluded because of non-representative titles and/or abstracts. Moreover, only studies written in English were included. Further, grey literature was not included, and this may have excluded some relevant studies that could have provided additional insight. In addition, no manual searches (i.e., via the reference lists of the included studies) were conducted. Although measurements were taken systematically and thoroughly during the abstraction and data extraction process, it is possible that objectivity was comprised in some way. However, efforts to restrict possible bias were conducted through inter-rater reliability testing, and we reached an agreement regarding the inclusion and exclusion criteria before and during the review process. Here, it is important to mention that the calculation of Cohen's kappa resulted in moderate agreement (0.57). This captures that it is quite a challenging task to include only studies with a qualitative methodology. Finally, it is possible that the decision to include only two raters made it easier to reach a consensus when disagreement occurred in the inclusion/exclusion process. Nevertheless, because of the large number of records being screened, especially in the early stages of the process, some mistakes may have occurred.

### Implications for practice and conclusion

This study identified the importance of professional development focusing on autism understanding and that there is still a lack of it which may cause a barrier to inclusive education regarding autistic students. This may provide leverage for school officials, especially school leaders/management, to implement professional development programs with autism-specific content. Because of the limited research on how to design inclusive instruction, a special didactic perspective is needed to support teachers’ understanding of the challenges in instruction autistic students encounter. The results demonstrate the need to combine the whole school structure, strategies, and attitudes with classroom-level strategies and content in a holistic approach are important assumptions for the practice. Although general data regarding inclusive education and attitudes towards inclusive education are important areas, more research is needed regarding academic and didactical perspectives. As presented in the introduction, inclusive education requires modifications in the whole school system in terms of structure, strategies, approaches, *and* pedagogical content (United Nations: General Comment Article 24, 2016), each of which should be equally important. Furthermore, in agreement with Macmillan et al. (2021) and Pellicano et al. (2014), we do identify that there is a need to focus on applied science to contribute to better outcomes for autistic students and to support the practice. To achieve this, it is important to strive to build bridges between research and practice (Guldberg, 2017).

### Note

The authors have chosen to use identity-first language i.e., autistic student/s. The is no consensus on the terminology but, several investigations have identified that autistic persons prefer identity-first (Bury et al., 2020; Kenny et al., 2016; Lei et al., 2021).

## Supplemental Material

sj-pdf-1-dli-10.1177_23969415221123429 - Supplemental material for Strategies in supporting inclusive education for autistic students—A systematic review of qualitative research results Click here for additional data file. Supplemental material, sj-pdf-1-dli-10.1177_23969415221123429 for Strategies in supporting inclusive education for autistic students—A systematic review of qualitative research results by Linda Petersson-Bloom and Mona Holmqvist in Autism & Developmental Language Impairments

sj-pdf-2-dli-10.1177_23969415221123429 - Supplemental material for Strategies in supporting inclusive education for autistic students—A systematic review of qualitative research results Click here for additional data file. Supplemental material, sj-pdf-2-dli-10.1177_23969415221123429 for Strategies in supporting inclusive education for autistic students—A systematic review of qualitative research results by Linda Petersson-Bloom and Mona Holmqvist in Autism & Developmental Language Impairments

sj-pdf-3-dli-10.1177_23969415221123429 - Supplemental material for Strategies in supporting inclusive education for autistic students—A systematic review of qualitative research results Click here for additional data file. Supplemental material, sj-pdf-3-dli-10.1177_23969415221123429 for Strategies in supporting inclusive education for autistic students—A systematic review of qualitative research results by Linda Petersson-Bloom and Mona Holmqvist in Autism & Developmental Language Impairments

sj-pdf-4-dli-10.1177_23969415221123429 - Supplemental material for Strategies in supporting inclusive education for autistic students—A systematic review of qualitative research results Click here for additional data file. Supplemental material, sj-pdf-4-dli-10.1177_23969415221123429 for Strategies in supporting inclusive education for autistic students—A systematic review of qualitative research results by Linda Petersson-Bloom and Mona Holmqvist in Autism & Developmental Language Impairments
