# Supplementary material for: Strategies in supporting inclusive education for autistic students—A systematic review of qualitative research results
Source: Autism Dev Lang Impair. 2022 Sep 21;7:23969415221123429. doi: 10.1177/23969415221123429 (PMC9620685; doi:10.1177/23969415221123429)
Supplement: sj-pdf-1-dli-10.1177_23969415221123429 - Supplemental material for Strategies in supporting inclusive education for autistic students—A systematic review of qualitative research results [file sj-pdf-1-dli-10.1177_23969415221123429.pdf]

Supplementary material Search Strategy

Full Search Strategy

1. ERIC (ProQuest)

Interface: ProQuest
Date of Search: 26 November 2018
Number of hits: 3,093

Field labels

- MAINSUBJECT.EXACT.EXPLODE = exploded subject heading
- MAINSUBJECT.EXACT non exploded subject heading
- TI,AB = title, abstract
- N/x = within x words, regardless of order
- * = truncation of word for alternate endings

(MAINSUBJECT.EXACT("Pervasive Developmental Disorders") OR TI,AB(autis* OR asperger* OR "childhood disintegrative" OR "pervasive development*" OR rett*))
AND
(MAINSUBJECT.EXACT.EXPLODE("School activities" OR ”Classes (Groups of Students)” OR "Educational Environment" OR "Educational Facilities" OR "Educational Methods" OR "Educational Practices" OR "Teacher Education" OR "Professional Development") OR MAINSUBJECT.EXACT("Class Size" OR "Educational Facilities Design" OR "Educational Strategies" OR "Instructional Design") OR TI,AB(((education* OR environment* OR learning OR school OR teaching OR instructional OR teacher*) N/3 (adaption* OR activit* OR facilit* OR method* OR design OR support* OR practice* OR provision* OR strateg* OR technique* OR intervention* or program*)) OR (environment* N/3 ("autism friendly" OR built OR condition* OR adaption* OR education* OR enabling OR learning OR support*)) OR "school class*" OR "class size*" OR classroom* OR "curricul* adaptation*" OR "group activit*" OR "group size*" OR grouping OR "teaching assistant*" OR "teacher aides" OR "satellite class*" OR "school schedul*" OR schoolyard* OR "school yard*" OR "evidence based" OR ((teacher* OR staff OR paraprofessional OR professional OR faculty OR staff) N/0 (development OR education OR training)) OR (autis* OR asperger* OR "childhood disintegrative" OR "pervasive development*")N/2 (awareness OR knowledge)))
AND
(((MAINSUBJECT.EXACT.EXPLODE("Children" OR "Adolescents") OR MAINSUBJECT.EXACT("Early Adolescents" OR "Preadolescents") OR TI,AB(child* OR adolescen* OR preadolescen* OR teen*)) AND (MAINSUBJECT.EXACT.EXPLODE("Education" OR "Schools") OR TI,AB(school* OR classroom* OR student*))) OR (MAINSUBJECT.EXACT("Mainstreaming" OR "Inclusion" OR "Middle School Students" OR "Middle Schools" OR "Elementary School Students" OR "Elementary Schools" OR "Compulsory Education") OR MAINSUBJECT.EXACT.EXPLODE("Secondary School Students" OR "Secondary Schools" OR "Elementary Secondary Education") OR TI,AB((elementary OR compulsory OR middle OR primary OR secondary) N/2 (school* OR education OR level)) OR ((inclusi* OR integrat* OR general OR regular) N/2 (adolescen* OR child* OR education OR school* OR student* OR pedagogy OR program OR provision* OR setting*)) OR college* OR "high school*" OR "public education" OR "public school" OR pupil* OR mainstream* OR "naturalistic setting*"))

2. Medline

Interface: Ovid
Date of Search: 3 December 2018
Number of hits: 1,046
Comment: In Ovid, two or more words are automatically searched as phrases; i.e. no quotation marks are needed

Field labels

- exp/ = exploded MeSH term
- / = non exploded MeSH term
- .ti,ab,kf. = title, abstract and author keywords
- adjx = within x words, regardless of order
- * = truncation of word for alternate endings

#	**Searches**	**Results**
1	exp Child Development Disorders, Pervasive/	30182
2	Rett Syndrome/	2371
3	(autis* or asperger* or childhood disintegrative or pervasive developmental or rett*).ti,ab,kf.	45992
4	or/1-3	49213
5	"Physical Education and Training"/	13169

6	Teacher Training/	141
7	Schools/	33727
8	exp Teaching/	79944
9	((education* or environment* or learning or school or teaching or instructional or teacher*) adj3 (adaption* or activit* or facilit* or method* or design or support* or practice* or provision* or strateg* or technique* or intervention* or program*)).ti,ab,kf.	196713
10	(environment* adj3 (autism friendly or built or condition* or adaption* or education* or enabling or learning or support*)).ti,ab,kf.	58050
11	((teacher* or staff or paraprofessional* or professional* or peer or faculty or staff) adj1 (development or education or training)).ti,ab,kf.	26454
12	((autis* or asperger* or childhood disintegrative or pervasive developmental) adj2 (awareness or knowledge)).ti,ab,kf.	182
13	(school class* or class size* or classroom* or curricul* adaptation* or group activit* or group size* or grouping or teaching assistant* or teacher aides or satellite class* or school schedul* or schoolyard* or school yard* or evidence based).ti,ab,kf.	136788
14	or/5-13	476630
15	Child/	1587949
16	Adolescent/	1897588
17	(child* or adolescen* or preadolescen* or teen* or youth).ti,ab,kf.	1492143
18	or/15-17	3172688
19	Schools/	33727
20	(school* or classroom* or student*).ti,ab,kf.	444486
21	or/19-20	448207
22	18 and 21	205404
23	"Mainstreaming (Education)"/	1028
24	((elementary or compulsory or middle or primary or secondary) adj2 (school* or education or level)).ti,ab,kf.	45147
25	((inclusi* or integrat* or general or regular) adj2 (adolescen* or child* or education or school* or student* or pedagogy or program or provision* or setting*)).ti,ab,kf.	24477
26	(college* or high school* or public education or public school or pupil* or mainstream* or naturalistic setting*).ti,ab,kf.	174028
27	or/22-26	372707
28	4 and 14 and 27	1046

3. Web of Science Core Collection

Interface: Clarivate Analytics<br>Date of Search: 26 November 2018<br>Number of hits: 4,044	Field labels<br>• TS/Topic = title, abstract, author keywords and Keywords Plus<br>• NEAR/x = within x words, regardless of order<br>• * = truncation of word for alternate endings

Set	Results	
# 4	4,044	#3 AND #2 AND #1<br>Indexes=SCI-EXPANDED, SSCI, A&HCI, CPCI-S, CPCI-SSH, ESCI Timespan=1945-2018
# 3	733,836	**TOPIC:** (((child* OR adolescen* OR preadolescen* OR teen* OR youth) AND (school* OR classroom* OR student*))) *OR* **TOPIC:** (((elementary OR compulsory OR middle OR primary OR secondary) NEAR/2 (school* OR education OR level))) *OR* **TOPIC:** (((inclusi* OR integrat* OR general OR regular OR ordinary) NEAR/2 (adolescen* OR

		child* OR education OR school* OR student* OR pedagogy OR program OR provision* OR setting*))) *OR* **TOPIC:** ((college* OR "high school*" OR mainstream* OR "public education" OR "public school" OR pupil* OR "naturalistic setting*")) Indexes=SCI-EXPANDED, SSCI, A&HCI, CPCI-S, CPCI-SSH, ESCI Timespan=1945-2018
# 2	6,043,750	**TOPIC:** (((education* OR environment* OR learning OR school OR teaching OR instructional OR teacher*) NEAR/3 (adaption* OR activit* OR facilit* OR method* OR design OR support* OR practice* OR provision* OR strateg* OR technique* OR intervention* or program*))) *OR* **TOPIC:** ((environment* NEAR/3 ("autism friendly" OR built OR condition* OR adaption* OR education* OR enabling OR learning OR support*))) *OR* **TOPIC:** ((classes OR "class size*" OR classroom* OR "curricul* adaptation*" OR "group activit*" OR "group size*" OR grouping OR "teaching assistant*" OR "teacher aides" OR "satellite class*" OR "school schedul*" OR schoolyard* OR "school yard*" OR "evidence based")) *OR* **TOPIC:** (((teacher* OR staff OR paraprofessional OR professional OR faculty OR staff) NEAR/0 (development OR education OR training))) *OR* **TOPIC:** (((autis* OR asperger* OR "childhood disintegrative" OR "pervasive development*")NEAR/2 (awareness OR knowledge))) Indexes=SCI-EXPANDED, SSCI, A&HCI, CPCI-S, CPCI-SSH, ESCI Timespan=1945-2018
# 1	72,163	**TOPIC:** ((autis* OR asperger* OR "childhood disintegrative" OR "pervasive development*" OR rett*)) Indexes=SCI-EXPANDED, SSCI, A&HCI, CPCI-S, CPCI-SSH, ESCI Timespan=1945-2018

5. Psycinfo

Interface: Ovid<br>Date of Search: 3 December 2018<br>Number of hits: 2,680<br>Comment: In Ovid, two or more words are automatically searched as phrases; i.e. no quotation marks are needed	Field labels<br>• exp/ = exploded controlled term<br>• / = non exploded controlled term<br>• .ti,ab,id. = title, abstract and author keywords<br>• adjx = within x words, regardless of order<br>• * = truncation of word for alternate endings

#	Searches	Results
1	exp autism spectrum disorders/	39217
2	exp Rett Syndrome/	825
3	(autis* or asperger* or childhood disintegrative or pervasive developmental or rett*).ti,ab,id.	49903
4	or/1-3	50275
5	learning environment/	9741
6	academic environment/	2166
7	classroom environment/	8298
8	classrooms/	15026
9	school environment/	11874
10	exp classroom management/	3227
11	exp school facilities/	19244
12	exp teaching/	113298
13	exp teacher education/	15799
14	paraprofessional education/	649
15	class size/	288
16	((education* or environment* or learning or school or teaching or instructional or teacher*) adj3 (adaption* or activit* or facilit* or method* or design or support* or practice* or provision* or strateg* or technique* or intervention* or program*)).ti,ab,id.	231240
17	(environment* adj3 (autism friendly or built or condition* or adaption* or education* or enabling or learning or support*)).ti,ab,id.	36144
18	((teacher* or paraprofessional* or professional* or peer or faculty or staff) adj1 (development or education or training)).ti,ab,id.	51822

19	((autis* or asperger* or childhood disintegrative or pervasive developmental) adj2 (awareness or knowledge)).ti,ab,id.	328
20	(school class* or class size* or classroom* or curricul* adaptation* or group activit* or group size* or grouping or teaching assistant* or teacher aides or satellite class* or school schedul* or schoolyard* or school yard* or evidence based).ti,ab,id.	140162
21	or/5-19	364082
22	(child* or adolescen* or preadolescen* or teen* or youth).ti,ab,id.	836255
23	(school* or classroom* or student*).ti,ab,id.	778351
24	schools/	28367
25	or/23-24	778561
26	22 and 25	230828
27	elementary schools/	7418
28	exp colleges/	15429
29	High Schools/	6431
30	junior high schools/	605
31	middle schools/	3897
32	elementary education/	3275
33	secondary education/	5864
34	high school education/	3795
35	middle school education/	695
36	exp elementary school students/	42179
37	Middle School Students/	8009
38	junior high school students/	11471
39	((elementary or compulsory or middle or primary or secondary) adj2 (school* or education or level)).ti,ab,id.	90341
40	"mainstreaming (educational)"/	6196
41	((inclusi* or integrat* or general or regular) adj2 (adolescen* or child* or education or school* or student* or pedagogy or program or provision* or setting*)).ti,ab,id.	29904
42	(college* or high school* or public education or public school or pupil* or mainstream* or naturalistic setting*).ti,ab,id.	332471
43	or/26-42	588163
44	4 and 21 and 43	2680
