# Supplementary material for: Strategies in supporting inclusive education for autistic students—A systematic review of qualitative research results
Source: Autism Dev Lang Impair. 2022 Sep 21;7:23969415221123429. doi: 10.1177/23969415221123429 (PMC9620685; doi:10.1177/23969415221123429)
Supplement: sj-pdf-2-dli-10.1177_23969415221123429 - Supplemental material for Strategies in supporting inclusive education for autistic students—A systematic review of qualitative research results [file sj-pdf-2-dli-10.1177_23969415221123429.pdf]

Supplementary material

Categories and Research Results

	Author/year	Cat	Outcomes of modification
26	Eman, 2014	NA	Theoretical frameworks can be helpful in improving educational policy and practice in regard to inclusion. The bio-ecological and transactional model can serve as a platform for exploring the inclusion of students with ASD in mainstream schools.
89	Sonnenmeier et al., 2005	NA	The study identified the potential benefit of improved collaborative teaming, clarification of learning goals, increased engagement, and added occasions for learning in the general education curriculum to improve augmentative and alternative communication outcomes for the student.
108	Young-Pelton & Doty, 2013	NA	The program improved teachers' skills to implement quality educational programs.
			School level
2	Anglim et al., 2018	A/V	The majority of participants felt that they lacked confidence in teaching and managing the behavior of students with ASD, and teachers perceived themselves as lacking resources, support, and training regarding working with students with ASD.
4	Ashbee & Guldberg, 2018	A/V	The biggest hindrances to inclusion were a lack of knowledge, stigma, and poor diagnostic practice. A rigid educational system and absence of shared understanding regarding inclusion also negatively impacted inclusion.
5*	Ashby, 2010	A/V	Teachers downplayed pupils' differences, and these differences were not dealt with in an open and honest way. Pupils with disabilities felt alienated in the classroom.
6	Barned et al., 2011	A/V	Participants held misconceptions about ASD but generally held positive attitudes regarding the inclusion of students with ASD in a regular classroom setting. However, attitudes toward inclusion were strongly influenced by the perceived severity of ASD.
7	Beecher & Darragh, 2011	A/V	Exposure to characters in books appears to positively affect attitudes and knowledge. Books have potential use in pre-service teacher education.
8	Bennett et al., 1996	A/V	Professionals must have an open mind and positive attitude and be willing to participate in additional training. Acknowledgement of parental involvement is essential in creating an inclusive environment for students with ASD. Administrators must support teachers via time and supplies.
9	Bond & Hebron, 2016	A/V	Tentative results show that Bronfenbrenner's model can be used as a framework for representing interactions within resource provisions schools. Staff at participating schools developed more confidence in their practice over time whilst identifying areas for progress.
10	Bond et al., 2017	A/V	Intervention increased skills and self-efficacy amongst staff regarding working with students with ASD. Broadening of organizational factors and increased collaboration.
11*	Bottema-Beutel et al., 2016	A/V	Participants rated recruitment of peers and family involvement as highly important, meeting with peers as medium highly important in creative an inclusive environment. Participants had diverse preferences in regard to intervention design and delivery.
12	Bottema-Beutel et al., 2017	A/V	Most children judged that failure to include peers with ASD was not acceptable. This perspective did not vary according to context or grade.
15*	Calder et al., 2013	A/V	High level of consistency between multiple informants, both in regard to the nature of friendships and the desire for friends, despite high variety in ASD characteristics of children. Social motivation of children was considered a key factor in inclusion, as were parents and teacher roles in supporting friendships among children.
16	Carter et al., 2014	A/V	Parents tended to be more satisfied with satellite support placement. Teaching practice and skills were identified as the most frequent facilitating factors across all respondents. Barriers to inclusion were generally related to individual characteristics of children such as anxiety or poor social skills.
18	Corkum et al., 2014	A/V	Staff found it difficult to meet the varying and wide-ranging needs of students with ASD, and perceived the need for multileveled and multipronged professional development, available as needed.
22*	Dillon et al., 2016	A/V	The qualitative data revealed differences in how the groups perceived group work, peers, and teaching staff. Peers were the biggest distraction faced in class for students with ASD.
23*	Dixon & Tanner, 2013	A/V	Both schools had difficulties implementing ongoing strategies. These difficulties were linked to an absence of collaboration amongst all stakeholders. The lack of student voices was evident and teachers and parents had some differing perspectives.
24	Eldar et al., 2010	A/V	Successful inclusion was characterized by a focus almost entirely placed on the included student, and was mostly in the social domain as opposed to the cognitive domain. Difficulties with inclusion were described in instances where focus was on both the student and environment. Coordinators ascribed success and difficulties to didactic

			aspects, environment, and student functioning, which included personal factors, stereotypical behavior, and individual abilities.
25	Emam & Farrell, 2009	A/V	Tensions appear to be shaped by ASD-related manifestations, especially social and emotional understanding. Tensions determine the quality of interactions, and inform the teacher's view regarding support needed by the student. Teachers rely on teacher's assistants to manage tensions.
28	Finch et al., 2013	A/V	Teachers require professional development to increase their understanding regarding ASD and to enhance their repertoire of teaching strategies.
29	Finke et al., 2009	A/V	Keeping lines of communication open and positively to attitudes about inclusion were identified as important factors.
30	Frederickson et al., 2010	A/V	Higher levels of staff knowledge, flexibility, and collaboration quality were more common in schools with resource bases. Low inclusion in mainstream lessons was identified as an issue. Schools could develop their resource base via staff training.
32	Glashan et al., 2004	A/V	Standard support services are too generic to accommodate all students with ASD. A complementary service with a greater focus on ASD (in terms of support for teachers and training) is needed.
34*	Guldberg et al., 2017	A/V	The knowledge co-creative process has three main aspects. 1) *Context of engagement* All schools in the study area were interested in participating but not all of them had sufficient resources. 2) *Empowerment and ownership.* The staff members with the most support from management and those who were most confident in their own skills were most willing and able to engage in co-construction. 3) *Voices and perspectives.* Although staff voices and perspectives were prominent, the experiences of children and their learning processes were part of the creation of stories.
36	Healy, 2011	A/V	Three themes emerged: *Understanding the student, supporting the demands of instruction*, which often related to academic roles during classroom instruction, and *providing behavior interventions*, which referred to positive interventions to support the student.
38	Higginson & Chatfield, 2012	A/V	Teachers exhibited increased positive attitudes towards students with ASD, who developed new strategies for interaction, leading to improved relationships with their parents.
40*	Humphrey & Lewis, 2008b	A/V	Barriers to learning exist in the school environment. Adaptations led to more positive experiences. Pupils reported that their needs were not sufficiently met.
42	Jindal-Snape, 2005	A/V	Parents emphasized that teachers, regardless of educational provision, should have autism-specific training. Regardless of provision, the quality of delivery, staff attitude, and curriculum modifications played an important role.
46	Kasa-Hendrickson & Kluth, 2005	A/V	The following principles were identified: Unconditional inclusion, making the classroom accessible, inclusion as a process, and working through challenges.
48*	Kucharczyk et al., 2015	A/V	Secondary schools do not sufficiently address the needs of pupils with ASD. Interventions should be both feasible and diverse, not "one size fits all". Professional development is needed to ensure that the staff is well prepared, in addition to basic ASD awareness and understanding for everyone working in school environments.
53	Lindsay et al., 2013	A/V	Identified challenges included understanding and managing behavior, the need for training, resources and policies, and creating an inclusive environment in the classroom. Teachers need resources and training to provide inclusive environments.
54	Lindsay et al., 2014	A/V	Strategies that led to a positive outcome included proper professional development training, resources and support for teachers, working in a team to optimize the best strategies, teaching methods tailored to the pupils' interests and ability, collaboration with parents, and a school climate of acceptance and awareness.
57	McCollow et al., 2013	A/V	The results were delivered as a case description and can be summarized in terms of conflicts from different individuals: teacher, principal, and parents. The needs of individual students must be considered when providing services that meet the federal requirements for appropriate education.
59	Maher, 2017	A/V	PE is viewed as an inclusive subject. However, teachers viewed teaching pupils with ASD and team games as challenges.
67*	Moyse & Porter, 2015	A/V	The results showed the personal adjustments that girls make in response to the hidden curriculum and the ways in which these go unnoticed, masking their need for support and contributing to underachievement in girls with ASD in school. Furthermore, some teachers misunderstand autism in girls, which contributes to the lack of support.
74*	Poon et al., 2014	A/V	The participants experienced difficulties in school. Programs that support peer and staff awareness may help to develop more supportive school environments.
75	Poonam, 2014	A/V	Participants agreed that social stories were effective in managing and modifying behaviors, particularly in combination with other strategies such as role-playing, visual support, praise. Lack of knowledge and preparation time were described as challenges.
77	Ravet, 2018	A/V	Both groups showed little or no knowledge regarding ASD and strategies for teaching students with ASD. Participants agreed that teachers need both of these to enable inclusion, and agreed that input regarding autism was insufficient in the ITE program,

			which was correlated with a lack of tutor expertise, worries about medical labeling, and questions about ITE curriculum overload and priorities.
78	Reupert et al., 2015	A/V	Participants had diverse views. Common themes that enabled inclusion were: *Acceptance,* children were accepted for who they are; *Transition issues and strategies; What works for my child,* safe place, school schedule and free time, flexibility, attractive social activities, and *Collaboration* between school and parents.
82	Sanahuja-Gavald et al., 2016	A/V	A positive commitment to inclusive education and collaborative support depends on the philosophy of inclusion and the educational level of schools.
83	Sansosti & Sansosti, 2012	A/V	Most participants had a positive attitude towards inclusion, but they also stated that full-time inclusion is not appropriate for all students, and that decisions should be made on an individual basis. Professional development was identified as vital for all staff to meet the needs of students with ASD.
86	Scheil et al., 2017	A/V	The overall experience was positive but participants identified some areas for improvement. The content of such programs must be examined prior to delivery and peer education should be developed or at least supervised by appropriately knowledgeable and trained school professionals or ASD specialists.
85	Sciutto et al., 2012	A/V	Identified themes: *Areas of Need,* greater understanding of ASD and individual needs; *Teacher qualities that had a positive impact,* tolerance, acceptance, and respect towards the child; *Strategies or practices that had a positive impact,* predictable environment, multiple ways for children to present knowledge, visual support, acceptance and understanding amongst peers.
90	Soto-Chodiman et al., 2012	A/V	Central themes: *Challenges;* increased workload and stress when trying to modify the curriculum and teaching techniques to meet the needs of students with ASD, teachers and peers found the behaviors of a student with ASD to be disconcerting. *Support;* the need to improve the level of school-based support and professional training for teachers and their Educational Assistance/Teacher Assistances, parents were identified as a source of important information about the student's needs.
91	Starr & Foy, 2010	A/V	Identified themes: The capability of school staff to efficiently manage the behavior of children with ASD, teacher education, and understanding regarding ASD. Collaboration between parents and school was identified as important.
92	Starr et al., 2016	A/V	Identified themes: *Relationship Building*; *Communication; Knowledge,* parents knowledge concerning the school system, legislation, and procedures, and the teachers' knowledge regarding teaching children with ASD; *Support,* participants reported that support could influence a successful transition outcome.
94	Symes & Humphrey, 2011a	A/V	Aspects of school culture that contribute to inclusion included: positive attitudes towards students with special educational needs, school leadership, support for inclusion, collaboration, and respect. Aspects that enable or hinder effective support: Access to expertise, communication, awareness of ASD amongst staff and teachers.
95	Symes & Humphrey, 2011b	A/V	The role of TAs was identified as helping students to stay focused and follow instructions from teachers. Most of the participants started work with no experience of ASD and received varying amounts of training, but all participants felt that general training about ASD was not what they needed. Lack of time for planning in collaboration with teachers was perceived as a key concern.
97	Taneja Johansson, 2014	A/V	Limited awareness of autism among school staff and varied views of stakeholders regarding the challenges faced by the child. School staff did not perceive social development as important, which differed from the other stakeholders' perceptions.
98	Teixeira De Matos & Morgado, 2016	A/V	Results were mostly consistent across information sources. Perception and acceptance of students with ASD was good amongst participants in theory. However, this did not translate into active behaviors in reality.
99	Tissot, 2011	A/V	Identified themes included: *Early diagnosis; Agreement regarding educational provision,* the stakeholders did not share views on these themes; *Finalizing the placement,* brought tension between the groups; *Working together,* was identified as an important but complex process from both perspectives.
100 *	Tobias, 2009	A/V	Effective support strategies were identified: Implementing good transition support, providing students with a mentor and meeting the needs of individuals, understanding and knowledge about ASD among staff, collaboration between school and parents, and balancing modifications of stressors within a busy school environment, whilst at the same time preparing students for the "real world".
101	Tso & Strnadová, 2017	A/V	The participants perceived that anxiety levels increased as their child transitioned from primary to high school. They reported varying levels of preparation and support during transition. The participants suggested that their knowledge of their child should be prioritized and used when planning the transition. However, only one parent clearly called for the students with ASD to be involved in the process.
107	Young et al., 2017	A/V	Teachers held positive views of inclusion, however their practice evidenced little in terms of agency to effect inclusion. They identified challenges related to external factors such as: Curriculum, lack of time, inadequate teacher education, lack of teacher training, large class sizes, and inadequate resources.

			School level
1	Able et al., 2015	A/V SoS	Peers need to be more informed about ASD to be more understanding. Participants identified that they needed more professional development.
17*	Cook et al., 2018	A/V SoS	Girls with ASD might perceive friendship differently than others, and many of the participants had trouble maintaining friendships. Although motivation regarding friendship was similar between autistic and non-autistic peers, girls faced many challenges related to social difficulties and masking of behaviors, which can be both a strength and hindrance. High absenteeism was reported.
19*	Daniel & Billingsley, 2010	A/V SoS	The participants had friends but described making friends as difficult. Challenges were identified as: suitable selection of friends, failing to initiate social contact, not wanting to bother others, and self-protection. They identified sharing interests and engaging in mutually enjoyable activities as important for maintaining friendships.
20*	Dann, 2011	A/V SoS	A large benefit of the specialized provision was the availability of assigned space for pupils. The following factors were perceived as important: structure and organization, making changes gradually, staff characteristics. In a school without a specialized program, training of staff and staff inexperience were issues. Pupils and their parents were also more concerned about friendship.
27*	Ezzamel et al., 2017	A/V SoS	An intervention led to positive changes regarding several social skills, increased emotion regulation, and an increased sense of empathy in one student. Peers of the student also demonstrated new skills, as well as an enhanced understanding and acceptance of the student's needs, and made a greater effort to include him.
35*	Hay & Winn, 2005	A/V SoS	Five themes were identified: *The nature of AS and its influence on behavior and social interactions, collaboration and relationships*, *burnout and lack of service, special educators work hard to assist others,* and *quality of school facilities*. The participants were supportive regarding inclusion but challenged by it at times. Teachers lacked skills, confidence, motivation, time, support, and resources to accommodate students with complex needs.
37*	Hedges et al., 2014	A/V SoS	Three themes emerged: *Inconsistencies* throughout the school day in terms of the timing of activities, and between home and school; *Difficulties with interpersonal connections* encompassed the challenges of students with ASD in developing and maintaining relationships and communication; *Knowledge/process breakdowns* were related to a lack of knowledge, preparation, and adequate support. A lack of attention to these themes made high school more challenging for students with ASD.
41*	Humphrey & Symes, 2010	A/V SoS	The largest difficulties/barriers for pupils with ASD in mainstream schools appear to arise from interactions with peers. Future work should focus on developing peer acceptance and understanding.
45	Kamps et al., 1998	A/V SoS	Peer interaction time increased during social programs and most of the typically developing peers enjoyed and benefitted from the peer group. The social intervention affected social participation for pupils with autism.
49*	Lamb et al., 2016	A/V SoS	Teachers should anticipate barriers that affect students with ASD in the field of PE, and provide strategies to facilitate communication and social interaction.
51	Landor & Perepa, 2017	A/V SoS	The resource base led to a positive perception of the social experience of schooling by participants.The base included: social skills training, a safe place, and class support. It was important that the entire school had awareness regarding ASD.
55	Locke et al., 2017	A/V SoS	The following implementation themes were identified: *Implementation process, staff, leadership* & *support.* In regard to specific social engagement interventions, *barriers* included time (most common barrier), implementation activities, and lack of resources, but *facilitators* included support for the principal and staff, open communication, and meetings with colleagues.
60	Majoko, 2016	A/V SoS	Reported benefits of mainstream schooling included access to mainstream education and opportunities for peer interactions. Challenges included bullying, self-imposed social isolation, social rejection, and transition periods.
61*	Marks et al., 2000	A/V SoS	Two areas were highlighted as important for students with ASD: 1) School/classroom climate, understanding and acceptance of peers, and 2) social skills training and social opportunities.
62*	McNerney et al., 2015	A/V SoS	Factors identified by parents as important included a nurturing, flexible, and inclusive environment that stressed both academic and life skills. Adolescents but not adults highlighted the importance of social relationships. Parents emphasized the anxiety and burden of choosing a secondary school.
66	McGillicuddy & O'Donnell, 2014	A/V SoS	Identified themes included: *Life is mainstream,* participants perceived that mainstream school is a challenge for students with ASD; *Much accomplished, much more to do,* teachers had a positive attitude regarding educating students with ASD, however more support is needed on an individual and system level; *Teacher self-perceptions of training, confidence, and expertise in teaching students with ASD,* experience is an important factor, however teacher education was not sufficient to prepare teachers for teaching students with ASD.

68*	O'Connor, 2016	A/V SoS	The child perceived increased self-esteem and social acceptance from peers increased. This positive shift (social acceptance) extended to other children who were previously socially isolated.
69*	O'Hagan & Hebron, 2017	A/V SoS	All students expressed an understanding and wish for friendship, all of them had experienced loneliness. The parents perceived the understanding of friendship to be at a more theoretical level, built around activities. Influences at an individual and contextual level involved factors such as levels of maturity, understanding of social rules, school environment, and peer acceptance.
76*	Potter, 2015	A/V SoS	The pupil showed a strong wish to have friends and thought that he had some friends. The pupil demonstrated some understanding regarding grades of friendship and exhibited a commitment to friendships over fairly long periods of time.
80*	Saggers et al., 2011	A/V SoS	Themes identified as having a positive or negative effect on learning and participation: *Teacher characteristics*; *Curriculum-related issues* (workload, handwriting, solutions to difficulties); *Support* (attitudes regarding specialist support, kinds of support); *Friendships* (attitudes towards socializing and loneliness); *Environmental considerations* (physical environment such as noise and crowding); *Teasing and bullying* (one of the most constraining factors for learning and participation).
81*	Saggers, 2015	A/V SoS	Identified themes: *Relations with peers,* positive relations with peers were identified as an enabling factor for inclusion; *Anxiety and stress in school,* factors in the environment (noise, crowds) and curriculum-related issues (homework, workload, handwriting) increased stress; *Working with teachers and other staff,* teacher characteristics was identified as an important factor in successful inclusion; *Negotiating difference,* constant need to negotiate difference within the mainstream setting.
87	Schultz et al., 2016	A/V SoS	The participants emphasized the need for collaboration with the school staff and parents. Parental advocacy and advocacy strategies were identified as important, as well as the role that parents can play in supporting their child's social development.
102 *	Turnbull et al., 2002	A/V SoS	Strategies at the universal and group level were not sufficient and needed to be addressed more individually to meet the needs of the pupil. The team collaborated to specify the strategies.
105 *	Wong, 2018	A/V SoS	Adolescents with ASD encounter a wide diversity of barriers and difficulties: Being bullied in school, learning English/mathematics, making and keeping friends, failure to advance in school. Further reform of policy and practice is needed to center the student when planning and implementing supportive services and educational programs.
			School level
13*	Bradley, 2016	SoS	Intervention positively affected pupils' self-esteem, increased social satisfaction, and decreased levels of bullying. Being a peer mentor increased the probability that pupils with autism felt that they could be a support to other peers.
21*	Dean et al., 2013	SoS	Autism-related behaviors manifest in inflexible interactions that systematically lead to exclusion.
44	Jones & Howley, 2010	SoS	The model showed positive outcomes for the children and was perceived to be effective by all participants, with a focus on increased staff confidence. Key features for achieving the aims of the model were identified as follows: Involvement of support teams, commitment of the school, partnership between parents and school staff.
47*	Keane et al., 2012	SoS	The program helped pupils with ASD to transition to inclusive educational settings. An ASD-specific curriculum was successful in preparing pupils with ASD. Teacher support, knowledge, provision of early interventions, and focus on social communications skills, as well as parental commitment and involvement, were considered by parents to be important factors.
50	Lamont, 2008	SoS	Three themes were identified as significant and positive: *Committed support team* (including parents, school staff, and consultant specialist), *issues and behaviors* examined in depth to create understanding, *flexible resourcing* to allow the support team to meet regularly.
52*	Lewis et al., 2005	SoS	The strategy may have benefited the pupil with ASD by encouraging appropriate social and academic skills. Selection of peer groups can be implemented in classroom settings.
56*	Lorenzo et al., 2013	SoS	IVA and highly structured and repetitive support tasks can improve executive functions and social skills in students with ASD. IVA has potential as a support tool in schools.
71*	Ochs et al., 2001	SoS	Positive inclusion seems to be facilitated by peer awareness of the abilities and difficulties of peers with HFA. Children whose conditions were explained to peers had wider ranges of friends. The practice of inclusion rests on peers, rather than teachers.
72	Peters, 2016	SoS	Social interaction and communication improved in the two children included in the study. However, changes in social interaction occurred at home and not at school.
79*	Rosetti & Goessling, 2010	SoS	Strategies that stood out: *Adults fade back,* prepares students for social interaction; *Prompt to be social,* look for forms of social interaction and prompt the student to successfully engage; and *Connect with peers,* engage peers to do some of the support that paraeducators do.
88	Smith et al., 2017	SoS	The qualitative data indicated that the primary intervention should address social interaction and instructional supports for the children. Implementation by teachers was

			inconsistent, and no reliable changes in academic development and independence were found. Community partnerships can help address and plan for interventions for children with ASD.
103	Wastney et al., 2007	SoS	Follow-up stories written by peers showed that they received new insights regarding the needs of student with ASD. The peers felt more confident regarding interaction and found more ways to support him. An added bonus was that many of the children spoke to their parents about the student, which made the parents more forthcoming to the child with autism.
106*	Woodfield & Ashby, 2016	SoS	The school had a problem-solving approach and worked in collaboration with parents. The school staff responded to the voices of the students. The students identified development of close relationships with their TAs as important. Social opportunities, frequency of adult support, and a predisposition for the school to function under the expectation that the students 'fit in' rather than essentially change the environment of the classrooms were identified as critical.
			Classroom level
3*	Asaro & Saddler, 2009	Did	The intervention had a positive effect on writing behaviors.
14*	Breivik & Hemmingsson, 2013	Did	Use of the device (ATD) contributed to positive attitudes towards writing. Use of the ATD had positive effects on writing development, which increased chances for participants to show knowledge and complete academic tasks.
31*	Gentry & Lindsey, 2012	Did	The student benefitted from the RR instruction practice. AT combined with best RR practices enhanced reading instruction when the student understood the purpose and use of the technology.
43	Jones & Block, 2006	Did	Collaboration should occur between PE teachers and the IEP-team, and regular meetings should take place with other teachers. Information about ASD should be given to the other children in the class. Visual support, modifications, and support should be available during PE. It may be helpful to minimize external stimuli, use equipment that provide extra stimulation, and allow breaks.
58*	Macdonald et al., 2017	Did	Ways to better fit both the research procedures and intervention to the context were developed. The intervention made in the classroom was not only feasible, but also resulted in a robust alignment between the research design and the environment.
70	Oakley et al., 2013	Did	In case study 1, understanding and engagement regarding the alphabet and phonetics increased during the intervention period. In case study 2, the intervention led to more positive attitudes towards reading and enhanced engagement in reading and writing.
93	Stokes et al., 2017	Did	*Strategies identified by principals*: Emphasis on the Individual Education Program (IEP) to improve teaching outcomes, teacher aides, assistive technology, strategies for communication, and the consistency and combination of approaches. *Strategies identified by teachers:* Visual support, structure, concrete instruction, timetables, and social stories. Unsuitable communication, disorganization, and a lack of understanding were identified as unhelpful in the classroom.
96*	Symes & Humphrey, 2012	Did	Students with ASD were included in lessons less efficiently compared with students from two other groups. Students with ASD were less likely to work independently and be socially included; this was most evident when a TA was present.
104*	Whalon & Hart, 2011	Did	As a result of the emphasis on learning to read in the early grades, reading instruction lacked a needed focus on comprehension. Therefore, *reading and language arts instruction did not always reflect or meet the individual needs of the learner with ASD.*
			Classroom level
39*	Howe & Stagg, 2016.	ES	Most participants reported that their sensory experiences affected their ability to learn. Schools need to conduct sensory profiles for each student with ASC.
63*	McAllister & Sloan, 2016	A/V ES	Noise was identified as a negative factor. The ASD 'Resource Base' was viewed as the students' 'home within the school', and was located away from what the students identified as the noisy parts of the school.
64	McAllister & Maguire, 2012a	ES	The 3D Classroom Design Kit facilitated the collaboration. ASD-specific considerations were identified.
65	McAllister & Maguire, 2012b	ES	Key design considerations included factors such as; a transitional buffet, designed cloakroom, consideration of pupils' sight lines when entering the classroom, provision for the positioning of visual support, high-level windows, provision of a quiet room directly accessible from the classroom and play area, a minimum of three workstations.
73	Pfeiffer et al., 2019	ES	Participation increased at home, school, and in the community. Examples of barriers: A dependence on headphones, stigma regarding use, and discomfort. Examples of benefits: Increased participation, decreased observed anxiety, improved attention, and enjoyment of tasks. Preparation for use was identified as a strategy to hinder barriers. Many of the children learned to predict when they needed to use the headphones.
84*	Santarosa & Conforto, 2016	ES	Laptops have a complex operational system and are not user friendly. Tablets are more user-friendly and can be helpful to students with ASD in several situations.

Note: *=perspective of student/s with ASD; Categories: NA=none applicable,Did = didactical perspective, A/V = attitudes and/or views, ES = environmental sensitivity, SoS = social skills focus).
