# Supplementary material for: Strategies in supporting inclusive education for autistic students—A systematic review of qualitative research results
Source: Autism Dev Lang Impair. 2022 Sep 21;7:23969415221123429. doi: 10.1177/23969415221123429 (PMC9620685; doi:10.1177/23969415221123429)
Supplement: sj-pdf-3-dli-10.1177_23969415221123429 - Supplemental material for Strategies in supporting inclusive education for autistic students—A systematic review of qualitative research results [file sj-pdf-3-dli-10.1177_23969415221123429.pdf]

Supplementary Material

Descriptive Characteristics of Articles Synthesized

	Author/Year	Country	SEND	Research Focus
1	Able et al., 2015.	USA		The social support needs of students with ASD in fully inclusive settings, and the teachers own need to facilitate students. Furthermore, the practices that teachers found to be successful.
2	Anglim et al., 2018.	Ireland		Self-efficacy of teachers in regard to inclusion and teaching students with ASD.
3	Asaro & Saddler, 2009.	USA		Intervention with a focus on self-regulated strategy development (SRSD), a method to improve writing skills.
4	Ashbee & Guldberg, 2018.	Palestine		Focus on concepts of inclusive education for students with ASD via a collaborative contextual enquiry method.
5	Ashby, 2010.	USA		Access to academic and social experiences for pupils, evaluated though an ableism lens. Efficacy and meaningful engagement for pupils with developmental disabilities such as ASD.
6	Barned et al., 2011.	USA		Pre-service teachers' knowledge and attitudes related inclusion of children with ASD.
7	Beecher & Darragh, 2011	USA		Do exposure to literature that portrays people with ASD enhances positive dispositions in pre-service teachers.
8	Bennett et al., 1996	USA		A collaborative case study focused on parents' views regarding successful inclusion.
9	Bond & Hebron, 2016.	UK		Perceptions of professionals regarding school resource provisions.
10	Bond et al., 2017	UK		Professional development in a resource provision school, staff perspectives.
11	Bottema-Beutel et al., 2016.	USA		Perceptions of youth with ASD regarding school-based, peer-mediated intervention programs.
12	Bottema-Beutel et al., 2017	USA		Pupils' perspectives and reasoning with respect to including peers with ASD.
13	Bradley, 2016	UK		Evaluating a peer-mentoring program for pupils with autism in secondary school, through the perspective of pupils with autism.
14	Breivik & Hemmingsson, 2013.	Sweden		Focus on how adolescents with AS experience writing in a school setting. Comparison of writing by hand vs. writing using a computerized Assistive Technology Device (ATD).
15	Calder et al., 2013	UK		Characteristics and perceptions of friendship in children with autism from the perspective of multiple types of informants.
16	Carter et al., 2014	Australia		Different perspectives on regular and support class placement and factors that can contribute to or obstruct inclusive education for pupils with ASD.
17	Cook et al., 2018	UK	Yes, partly	Experiences of learning, friendship, and bullying of girls with ASD from the perspectives of the girls and their parents.
18	Corkum et al., 2014	Canada		Identifying professional development needs for staff working with children with ASD in an inclusive school environment.
19	Daniel & Billingsley, 2010.	USA		Perspectives of boys with ASD on establishing and maintaining friendships.
20	Dann, 2011	UK		Experience of the transition to secondary school (mainstream school with special provisions for ASD or regular mainstream school) from three different perspectives.
21	Dean et al., 2013	USA		The narratives of a girl with autism and her friends at a mainstream school.
22	Dillon et al., 2016	UK		Experiences of mainstream school from the perception of children with ASD compared with typically developed peers.
23	Dixon & Tanner, 2013	Australia		Experiences of the transition to high school in two students, perceptions of multiple stakeholders.
24	Eldar et al., 2010.	Israel		Inclusion of children with ASD through the perspectives of inclusion coordinators.
25	Emam & Farrell, 2009.	UK		Exploration of tensions that teachers may experience in educating students with ASD, and how these might shape support given.

26	Eman, 2014	UK		Inclusion of students with ASD, examined from developmental systems perspectives such as the bio-ecological and transactional models.
27	Ezzamel et al., 2017	UK		Peer-meditated intervention. Perceptions of a peer-meditated network and its impact by a pupil with ASD, peers, and staff.
28	Finch et al., 2013	USA		Perspectives on the needs of pupils with ASD in an education setting.
29	Finke et al., 2009	USA		Understanding strategies that teachers use for the inclusion of children with ASD who use Augmentative Alternative Communication (ACC).
30	Frederickson et al., 2010	UK		Exploring the characteristics of mainstream education placements for pupils with ASD in schools with and without an ASD resource base, through the perspective of staff members.
31	Gentry & Lindsey, 2012	USA		An intervention including individualized fiction-reading instruction that focused on Real Reading (RR) instruction with assistive technologies (AT).
32	Glashan et al., 2004	Scotland, UK	Yes, partly	Teacher experiences of support for pupils with autism.
33	Grenier & Yeaton, 2011	UK		How previewing can be a strategy for physical education.
34	Guldberg et al., 2017	UK	Yes, partly	Participatory research with a focus on collective enquiry through participant experiences, knowledge, and use of technology-enhanced learning. Emphasis on eliciting reflection rather than a focus on change and action.
35	Hay & Winn, 2005	Australia		Educational issues affecting secondary students with AS from the perspectives of general teachers, special education teachers, students with AS, and their parents.
36	Healy, 2011	USA		Perspectives of paraeducators regarding workplace demands.
37	Hedges et al., 2014	USA		Multiple perspectives on challenges impacting the success of students with ASD in high school.
38	Higginson & Chatfield, 2012.	New Zealand		ASD professional development program for teachers in schools that participated in an ASD project.
39	Howe & Stagg, 2016	UK		Experiences of sensory issues in students with ASD.
40	Humphrey & Lewis, 2008b	UK		Views and experiences of mainstream school by pupils with AS.
41	Humphrey & Symes, 2010	UK		Views of pupils with ASD on the role of social support in determining their response to bullying. Identification of barriers to the development of social support during bullying.
42	Jindal- Snape, 2005	Scotland, UK		Perceptions of professionals and parents on educational provisions, support in provisions, and what does and does not work.
43	Jones & Block, 2006	USA	Yes	Successful methods and techniques used to include a student in PE class.
44	Jones & Howley, 2010	UK	Yes	Investigation of an interaction program (promoting inclusion) and the outcome for participating children. The impact of the program for trainees and schools, as well as the key features of this system for delivering training, were examined.
45	Kamps et al., 1998	USA	Yes, partly	Focus on perceptions of peers without ASD. Peers were involved in school-based integrated activities with students with autism.
46	Kasa-Hendrickson & Kluth, 2005	USA	Yes	Experiences of teachers practicing inclusion and seeking strategies to provide access and opportunity to students with ASD in a general education classroom.
47	Keane et al., 2012	Australia		Focus on whether a satellite program achieved its goal of ongoing placement for a more inclusive educational setting. Presentation of information regarding pupils' achievements, adaptive functioning, and quality of life.
48	Kucharczyk et al., 2015	USA		Perspective on high school education from multiple perspectives.
49	Lamb et al., 2016	UK		Views of children with ASD on physical education.
50	Lamont, 2008	New Zealand		Participatory action study with a focus on identifying the most effective strategies in diverse settings to increase reciprocal

			social interactions, and to identify the tools and resources that support this process.
51	Landor & Perepa, 2017	UK	Case study examining whether resource bases enable social inclusion of pupils with ASD. From the perspective of different stakeholders.
52	Lewis et al., 2005	UK	Examination of whether collaborative group work using a computer could facilitate inclusion of a pupil with ASD.
53	Lindsay et al., 2013	Canada	Teachers' perceptions of the challenges in creating an inclusive classroom environment for pupils with ASD.
54	Lindsay et al., 2014	Canada	Teachers' perceptions of strategies for including pupils with ASD in mainstream classrooms.
55	Locke et al., 2017	USA	Perspectives of school staff regarding implementing a social engagement intervention for children with autism. Areas of focus were school factors that affect the general implementation of evidence-based practices, experiences of implementing a social engagement intervention, and challenges and supports in implementing the intervention.
56	Lorenzo et al., 2013	Spain	Use of Immersive Virtual Reality (IVR) as a support and learning tool.
57	McCollow et al., 2013.	USA	A school district implemented a research-based model into the school system to deliver support to teachers. The model contained technical assistance teams intended to provide continuing professional development to general and special educators.
58	Macdonald et al., 2017	Australia	Participatory research regarding an intervention to improve the inclusivity of students with ASD in mainstream schools and to establish a method for evaluating program effectiveness.
59	Maher, 2017	UK	Para-professionals' views regarding the inclusion of students with ASD in physical education.
60	Majoko, 2016	Zimbabwe	Teacher perceptions of inclusion of students with ASD in terms of challenges and supports.
61	Marks et al, 2000	USA	Building portraits of adolescents with AS and their school experiences.
62	McNerney et al., 2015.	UK	Factors that directly influence secondary school choice for adolescents with autism from the perspectives of multiple informants.
63	McAllister & Sloan, 2016	UK	Examination of an autism-friendly school environment from the perspective of adolescents with ASD.
64	McAllister & Maguire, 2012a	Northern Ireland	Examining the ASD Classroom Design Kit in facilitating collaboration between teachers and architects.
65	McAllister & Maguire, 2012b	Northern Ireland	Highlighting the 16 design considerations identified in the previous study.
66	McGillicuddy & O'Donnell, 2014	Republic of Ireland	Teacher perceptions of inclusive education for students with ASD at the post-primary level.
67	Moyse & Porter, 2015	UK	How the hidden curriculum affects girls with ASD and impacts their experiences of school.
68	O'Connor, 2016	USA	Examining the use of circles of friends. Focus on changing attitudes amongst peers in relation to children with AS.
69	O'Hagan & Hebron, 2017	UK	Friendship amongst adolescents with ASD, with a focus on the meaning and nature of friendship and factors that influence the development of friendship.
70	Oakley et al., 2013	Australia	How Information and Communication Technologies (ICTs) can support the creation of multimodal texts.
71	Ochs et al., 2001	USA	Demonstrating the social realities of inclusion in 16 students with autism.
72	Peters, 2016	Hong Kong	Participation and engagement of children with ASD during use of a Social Communication and Interaction model.
73	Pfeiffer et al., 2019	USA	Parent and teacher perceptions of benefits and limitations of using noise-attenuating headphones for children with ASD.
74	Poon et al., 2014	Singapore	Student perspectives of their secondary school experiences.

75	Poonam, 2014	USA		Teacher experiences regarding the creation of social stories and perspectives regarding the impact of social stories on students with ASD.
76	Potter, 2015	UK	Yes	Perspectives of a student with ASD regarding friendship.
77	Ravet, 2018	UK		Student and tutor perceptions of autism education with respect to a four-year Initial Teacher Education (ITE) program.
78	Reupert et al., 2015	Australia		Parent views regarding factors that enable inclusion.
79	Rosetti & Goessling, 2010	USA		Strategies of paraeducators in facilitating friendship.
80	Saggers et al., 2011	Australia		Experiences of inclusivity in high school, from the view of students with ASD. Recognizing practices that facilitate and constrain the learning and participation.
81	Saggers, 2015	Australia		Student perspectives on how to improve inclusivity for those with ASD in high school education.
82	Sanahuja-Gavald et al., 2016	Spain		The inclusion process for students with ASD by focusing on support organization, collaboration, and inclusive methodologies.
83	Sansosti & Sansosti, 2012	USA		Analysis of definitions and decision-making considerations regarding ASD for a school district.
84	Santarosa & Conforto, 2016	Brazil	Yes	Focus on the relationship between students with ASD and mobile devices.
85	Sciutt et al., 2012	USA		School-related challenges and instructional practices through the perspectives of people with AS and their caregivers.
86	Scheil et al., 2017	USA		Evaluation of student impressions of the Kit for Kids (KfK) program, which is a peer educational program.
87	Schultz et al., 2016	USA		Teacher perceptions of parent-professional collaboration with a focus on helpful parental involvement and advocacy strategies to guarantee the success of students in inclusive settings.
88	Smith et al., 2017	USA		Participatory research with the goal of developing and evaluating interventions for children with autism.
89	Sonnenmeier et al., 2005	USA	Yes	Implementation of a team-support-planning model (Beyond Access Model) for a student with autism.
90	Soto-Chodiman et al., 2012	Australia		Experiences of teachers who have had a student with ASD in their classroom.
91	Starr & Foy, 2010	Canada		Parent perspectives regarding the education of their child with ASD.
92	Starr et al., 2016	Canada		Perceptions of parents, teachers, and early intervention service providers regarding the transition to kindergarten for children with ASD.
93	Stokes et al., 2017	Australia		Teaching strategies found to be successful by teachers and principals for their students with High functioning ASD.
94	Symes & Humphrey, 2011a	UK		Perceptions of Teacher's Assistants (TAs) regarding aspects of school culture that contribute to the development of inclusion.
95	Symes & Humphrey, 2011b	UK		Examination of how TAs support students, perceive effects of professional training, and view their relationships with teachers. Identification of factors that facilitate or constrain their practice.
96	Symes & Humphrey, 2012	UK		Examination of whether students with ASD were effectively included in lessons, compared with students with dyslexia or those with no Special Educational Needs, and an examination of how the presence of a TA influences inclusion.
97	Taneja Johansson, 2014	India		Awareness of autism in stakeholders and their perspectives on children with autism.
98	Teixeira De Matos & Morgado, 2016	Portugal		Examination of how participation transpires in students with ASD in mainstream school.
99	Tissot, 2011	UK	Yes	Views of parents and local authorities regarding how they perceive and experience the process of shaping educational provisions for students with ASD.
100	Tobias, 2009	UK		Additional support at secondary school, as perceived by students with ASD and their parents.
101	Tso & Strnadová, 2017	Australia		Experiences of parents of students with ASD in regard to the transition to high school and the collaboration between support at home and at school.

102	Turnbull et al., 2002	USA	Implementation of positive behavior support (PBS) on universal, group, and individual support levels, with a focus on one student.
103	Wastney et al., 2007	New Zealand	To facilitate acceptance and understanding amongst peers regarding autism, parents explained their child's challenges and strengths to his new classmates.
104	Whalon & Hart, 2011	USA	Experience reading instructions in children with ASD.
105	Wong, 2018	Hong Kong	Self-portrayals of 17 adolescents, who reveal challenges they have encountered.
106	Woodfield & Ashby, 2016	USA	Experiences of high school students who type to communicate and the support systems around them.
107	Young et al., 2017	Ireland	Examination of teachers' attitudes regarding inclusion, initial teacher education, professional development opportunities, and their experiences working with students with ASD in inclusive settings.
108	Young-Pelton & Doty, 2013	USA	Evaluating the Montana Autism Education Project (MAEP) with a focus on increasing knowledge regarding educational practices for students with ASD.
