# Supplementary material for: Strategies in supporting inclusive education for autistic students—A systematic review of qualitative research results
Source: Autism Dev Lang Impair. 2022 Sep 21;7:23969415221123429. doi: 10.1177/23969415221123429 (PMC9620685; doi:10.1177/23969415221123429)
Supplement: sj-pdf-4-dli-10.1177_23969415221123429 - Supplemental material for Strategies in supporting inclusive education for autistic students—A systematic review of qualitative research results [file sj-pdf-4-dli-10.1177_23969415221123429.pdf]

Supplementary Material

References, not referred to in the text

*Bennett, T., Rowe, V., & DeLuca, D. (1996). Getting to know Abby. *Focus on Autism and Other Developmental Disabilities*, *11*(3), 183–188. https://doi.org/10.1177/108835769601100308

*Bottema-Beutel, K., Turiel, E., DeWitt, M. N., & Wolfberg, P. J. (2017). To include or not to include: Evaluations and reasoning about the failure to include peers with autism spectrum disorder in elementary students. *Autism*, *21*(1), 51–60. https://doi.org/10.1177/1362361315622412

*Bradley, R. (2016), 'Why single me out?' Peer mentoring, autism and inclusion in mainstream secondary schools. *British Journal of Special Education*, *43*, 272–288. https://doi.org/:10.1111/1467-8578.12136

*Dann. R. (2011) Secondary transition experiences for pupils with Autistic Spectrum Conditions (ASCs). *Educational Psychology in Practice*, *27*(3), 293–312. https://doi.org/10.1080/02667363.2011.603534

*Dean, M., Adams, G. F., & Kasari, C. (2013). How narrative difficulties build peer rejection: A discourse analysis of a girl with autism and her female peers. *Discourse Studies*, *15*(2), 147–166. https://doi.org/10.1177/1461445612471472

*Dixon, R. M., & Tanner, K. (2013). The Experience of Transitioning Two Adolescents with Asperger Syndrome in Academically Focused High Schools. *Australasian Journal of Special Education*, *37*(1), 28–48. https://doi.org/10.1017/jse.2013.5

*Emam, M. M. (2014). The closeness of fit: Towards an ecomap for the inclusion of pupils with ASD in mainstream schools. *International Education Studies*, *7*(3), 112–125.

*Finke, E.H., McNaughton, D.B., & Drager, K.D.R. (2009). "All children can and should have the opportunity to learn": General education teachers' perspectives on including children with autism spectrum disorder who require AAC. *AAC: Augmentative & Alternative Communication*, *25*(2), 110–122. https://doi.org/10.1080/07434610902886206

*Healy, C. (2011). One-to-one in the inclusive classroom: The perspectives of paraeducators who support adolescents with autism spectrum disorder. *Journal of the American Academy of Special Education Professionals*, 77- 92.

*Kamps, D. M., Kravits, T., Lopez, A. G., Kemmerer, K., Potucek, J., & Harrell, L. G. (1998). What do the peers think? Social validity of peer-mediated programs. *Education and Treatment of Children, 21*(2), 107–134.

*Kasa-Hendrickson, C., & Kluth, P. (2005). "We have to start with inclusion and work it out as we go": Purposeful inclusion for non-verbal students with autism. *International Journal of Whole Schooling, 2*(1), 2–14.

*Lamont, R. (2008). Learning from each other: The benefits of a participatory action research project on the culture, activities and practices of the adults supporting a young child with autism spectrum disorder. *Kairaranga, 9*, 38–42.

*Landor, F., & Perepa, P. (2017). Do resource bases enable social inclusion of students with Asperger syndrome in a mainstream secondary school? *Support for Learning*, 32(2).

*Locke, J., Harker, C., Wolk, C. B., Shingledecker, T., Barg, F., Mandell, D., Beidas, R. (2017). Pebbles, rocks, and boulders: The implementation of a school-based social engagement intervention for children with autism. *Autism, 21*(8), 985–994. https://doi.org/10.1177/1362361316664474

*Lorenzo, G., Pomares, J., & Lledó, A. (2013). Inclusion of immersive virtual learning environments and visual control systems to support the learning of students with Asperger syndrome. *Computers & Education*, *62*, 88–101. https://doi.org/10.1016/j.compedu.2012.10.028

*Majoko, T. (2016). Inclusion of children with autism spectrum disorders: Listening and hearing to voices from the grassroots. *Journal of Autism & Developmental Disorders, 46*(4), 1429–1440. https://doi.org/10.1007/s10803-015-2685-1

*McGillicuddy, S., & O'Donnell, G. M. (2014). Teaching students with autism spectrum disorder in mainstream post-primary schools in the Republic of Ireland. *International Journal of Inclusive Education, 18*(4), 323–344. https://doi.org/10.1080/13603116.2013.764934

*McNerney, C., Pellicano, E., & Hill, V. (2015). Choosing a secondary school for young people on the autism spectrum: A multi-informant study. *International Journal of Inclusive Education, 19(*10), 1096–1116. https://doi.org/:10.1080/13603116.2015.1037869

*O'Connor, E. (2016). The use of 'Circle of Friends' strategy to improve social interactions and social acceptance: A case study of a child with Asperger's Syndrome and other associated needs. *Support for Learning, 31*, 138–147. https://doi.org/10.1111/1467-9604.12122

*Peters, B. (2016). A model for enhancing social communication and interaction in everyday activities for primary school children with ASD. *Journal of Research in Special Educational Needs, 16*(2), 89–101. https://doi.org/10.1111/1471-3802.12059

*Potter, C. (2015). "I didn't used to have much friends": Exploring the friendship concepts and capabilities of a boy with autism and severe learning disabilities. *British Journal of Learning Disabilities, 43*(3), 208–218. https://doi.org/ 10.1111/bld.12098

*Sanahuja, G. J. M., Olmos, R. P., & Morón, V. M. (2016). Collaborative support for inclusion. *Journal of Research in Special Educational Needs, 16*, 303–307. https://doi.org/10.1111/1471-3802.12293

*Scheil, K. A., Campbell, J. M., & Bowers-Campbell, J. (2017). An initial investigation of the Kit for Kids peer educational program. *Journal of Developmental and Physical Disabilities, 29*(4), 643–662. https://doi.org/10.1007/s10882-017-9540-6

*Schultz, T. R., Able, H., White, T., & Sreckovic, M. A. (2016). Parent-teacher collaboration: Teacher perceptions of what is needed to support students with ASD in the inclusive classroom. *Education and Training in Autism and Developmental Disabilities, 51*(4), 344–354. http://www.jstor.org/stable/26173862

*Smith, T., Iadarola, S., Mandell, D. S., Harwood, R., & Kasari, C. (2017). Community-partnered research with urban school districts that serve children with autism spectrum disorder. *Academic Pediatrics, 17*(6), 614.

*Sonnenmeier, R. M., McSheehan, M., & Jorgensen, C. M. (2005). A case study of team supports for a student with autism's communication and engagement within the general education curriculum: Preliminary report of the beyond access model. *AAC: Augmentative and Alternative Communication, 21*(2), 101–115. https://doi.org/10.1080/07434610500103608

*Teixeira De Matos, I., & Morgado, J. (2016). School participation of students with autism spectrum disorders. *Journal of Research in Special Educational Needs, 16,* 972–977. https://doi.org/10.1111/1471-3802.12240

*Turnbull, A., Edmonson, H., Griggs, P., Wickham, D., Sailor, W., Freeman, R., Guess, D. (2002). A blueprint for schoolwide positive behavior support: Implementation of three components. *Exceptional Children, 68*(3), 377. https://doi.org/10.1177/001440290206800306

*Young-Pelton, C. A., & Doty, D. (2013). Improving educational programs for students with autism in rural schools: A preliminary program description of the Montana Autism Education Project. *Rural Special Education Quarterly, 32*(3), 24–32. https://doi.org/10.1177/875687051303200305
